# The role of intra-abdominal pressure in human testicular migration

**DOI:** 10.1590/S1677-5538.IBJU.2021.99.03

**Published:** 2020-11-18

**Authors:** Natasha T. Logsdon, Francisco J.B. Sampaio, Luciano Alves Favorito

**Affiliations:** 1 Universidade do Estado do Rio de Janeiro Unidade de Pesquisa Urogenital Rio de JaneiroRJ Brasil Unidade de Pesquisa Urogenital, Universidade do Estado do Rio de Janeiro – Uerj, Rio de Janeiro, RJ, Brasil

**Keywords:** Cryptorchidism, Intra-Abdominal pressure, Prune Belly Syndrome

## Abstract

**Objectives:**

This review aims to study the role of the abdominal wall in testicular migration process during the human fetal period.

**Materials and Methods:**

We performed a descriptive review of the literature about the role of the abdominal wall in testicular migration during the human fetal period.

**Results:**

The rise in intra-abdominal pressure is a supporting factor for testicular migration. This process has two phases: the abdominal and the inguinal-scrotal stages. The passage of the testis through the inguinal canal occurs very quickly between 21 and 25 WPC. Bilateral cryptorchidism in Prune Belly syndrome is explained by the impaired contraction of the muscles of the abdominal wall; mechanical obstruction due to bladder distention and structural alteration of the inguinal canal, which hampers the passage of the testis during the inguinoscrotal stage of testicular migration. Abdominal wall defects as gastroschisis and omphaloceles are associated with undescended testes in around 30 to 40% of the cases.

**Conclusions:**

Abdominal pressure wound is an auxiliary force in testicular migration. Patients with abdominal wall defects are associated with undescendend testis in more than 30% of the cases probably due to mechanical factors; the Prune Belly Syndrome has anatomical changes in the anterior abdominal wall that hinder the increase of intra-abdominal pressure which could be the cause of cryptorchidism in this syndrome.

## INTRODUCTION

Testicular descent is a complex and multifactor event and has great importance for testicular development and the comprehension of cryptorchidism. The moment when testicular migration begins is controversial but in human embryological development the testes descend from the abdomen to the scrotum, traversing the abdominal wall and the inguinal canal (1–4).

The most accepted theories to explain the testicular migration are: (a) rise of intra-abdominal pressure (5); (b) development of the structures near the testis (epididymis, spermatic vases and deferent ducts) (6, 7); (c) the stimulus originated in the genitofemoral nerve (8); (d) hormonal stimulus originated in the placental gonadotrophin and the testosterone produced by the fetal testes (8–12); and (e) the gubernaculums development (1, 3). Studies about the role of abdominal wall and the intra-abdominal pressure in the testicular descent are scarce and this topic is controversial. In this review we will describe the role of the intra-abdominal pressure in the testicular migration process and will analyze some aspects of the abdominal wall defects (AWD) and the implications of these anomalies in testicular migration.

## MATERIALS AND METHODS

In this study we carried out a review about the role of the intra-abdominal pressure in testicular migration during the human fetal period and abdominal wall defects and testicular migration and undescended testis. We analyzed papers published in the past 50 years in the databases of Pubmed, Embase and Scielo, using the key expressions “abdominal wall”; “intra-abdominal pressure”; “abdominal wall defects”; “undescended testis” and “testicular migration”. In this review we found several papers in these databases and we included only papers in English and excluded case reports, editorials and opinions of specialists.

## RESULTS

**Figure 1:**
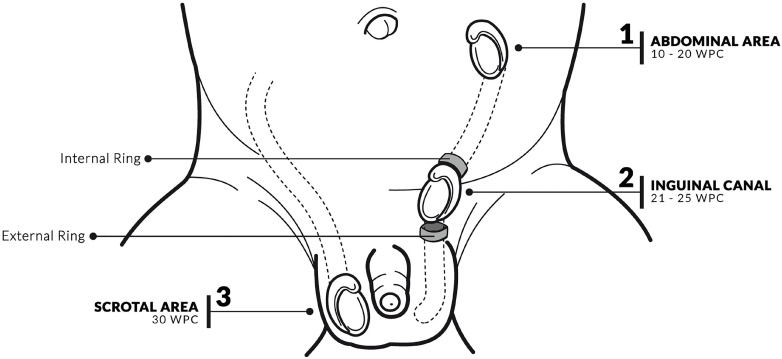
Schematic drawing showing the testicular migration during human fetal period. We can observe the testis situated in abdominal position between 10 and 20 weeks post conception (WPC) (1); in inguinal position (testis situated between internal and external inguinal rings) during 21 and 25 WPC (2) and in scrotal position after the 30WPC (3).

Testicular migration has two phases: the abdominal and the inguinal-scrotal stages (1–3) (Figure-1). During the abdominal stage the testis migrates from the abdomen to the internal inguinal ring. This process begins around the 8th WPC and lasts until the 15th WPC. During the eighth week of gestation, the testis and mesonephros are linked to the posterior abdomen wall by a peritoneal fold (1). The portion of this fold called the diaphragmatic ligament degenerates, turning into the cranial portion of the gonadal mesentery. This structure is called the caudal gonadal ligament, which gives rise to the gubernaculum testis (3, 13). One of the factors involved in cryptorchidism is the failure of the gubernaculum to migrate all the way to the scrotum (14, 15). The influence of fetal androgens on the fetal gubernaculum's development is very important for the alterations of this structure, and the changes in its secretions can be one of the factors involved in cryptorchidism (16).

**Figure 2:**
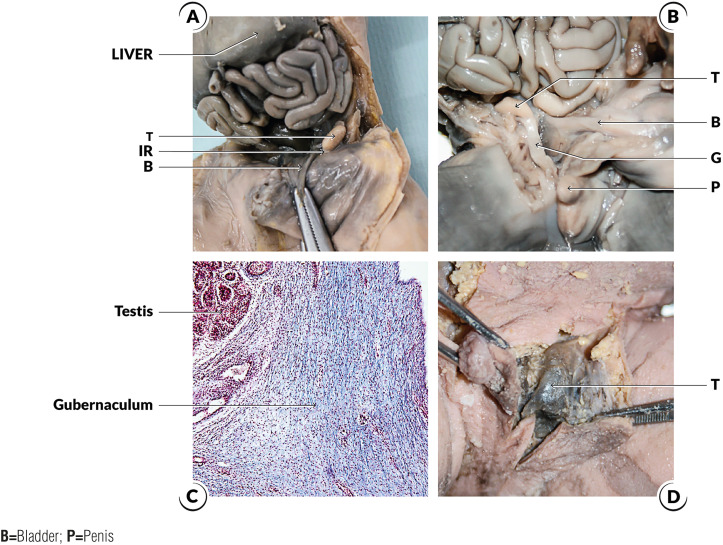
The figure shows the steps of testicular migration during the human fetal period. A) The figure shows a male fetus with 15 weeks post-conception with both testes situated in the abdomen. The abdominal wall was dissected to show the position of the testis (T) above the internal ring (arrowhead); B) The figure shows a male fetus with 18 weeks post-conception. The abdominal wall was dissected and we can observe the right testis (T) just above the internal ring (IR) and the distal insertion of the gubernaculum testis (G); C) Photomicrography of the same fetuses of Figure 2B showing the proximal insertion of gubernaculum testis. We can observe that the gubernaculum is attached to the testis. Masson's trichrome X100; and D) The figure shows a male fetus with 30WPC with both testes situated in the scrotum, we can observe the left testis (T) in scrotal position.

The second stage of testicular migration (inguinal-scrotal stage) is the transition of the testes through the inguinal canal until their definitive arrival in the scrotum (Figure-2) (1, 17). Distally the gubernaculum approaches the inguinal region. At this moment, the future inguinal canal is still only a space in the musculature of the anterior abdominal wall, where only mesenchyme tissue exists. In this region, the genital branch of the genitofemoral nerve crosses the abdominal wall and descends to the scrotum where it will innervate the cremaster muscle, and subsequently, in the caudal to cranial direction, will provide the nerve supply to the gubernaculum (1, 18, 19). During this stage, after the testis crosses the external inguinal ring the gubernaculum migrates across the pubic region to reach the scrotum. In rodents, the active proliferation of the gubernacular tip and cremaster muscle, the muscle's rhythmic contraction, and the chemotactic gradient provided by the CGRP altogether result in migration of the testes into the scrotum. The importance of this mechanism is corroborated by experimental models where the sectioning of the genitofemoral nerve leads to cryptorchidism (19–21).

The passage of the testis through the inguinal canal occurs very quickly between 21 and 25 WPC (1–4). In a recent paper with more than 240 human male fetuses studied shows that all the fetuses older than 30 weeks already had the testes in the scrotum (22). Other authors, however, report that the testicular migration is only completed after the 32nd week post-conception (1–3).

## RISE IN INTRA-ABDOMINAL PRESSURE AND TESTICULAR MIGRATION

An old and quite controversial theory of testicular migration is the role of intra-abdominal pressure. The contraction of the abdominal wall musculature, the growth of the liver and intestines, as well as the accumulation of meconium increase the pressure inside the fetal abdomen, which according to some authors would favor testicular migration (4, 17). Another fact that speaks in favor of this theory is the high incidence of cryptorchidism in patients with abdominal wall defects, such as omphaloceles, gastroschisis and Prune Belly Syndrome (23, 24). This theory, however, does not explain cases of asymmetry in testicular migration, where one testis migrates normally, while the other is located in the inguinal canal or abdomen (25).

**Figure 3:**
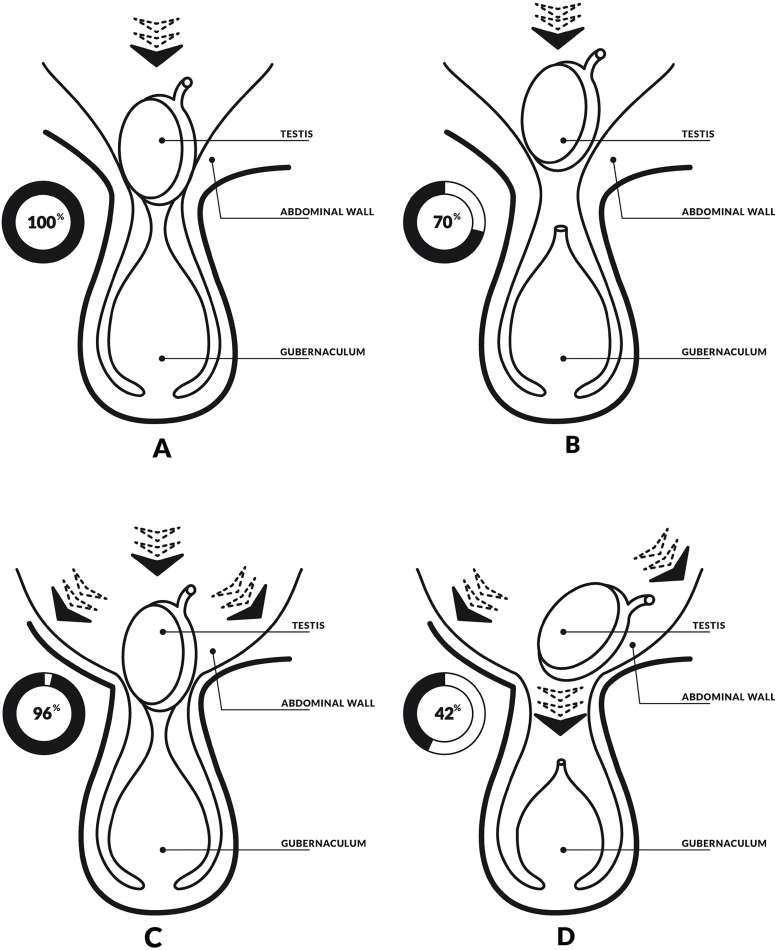
Schematic drawing, based on experimental paper of Attah and Hutson (4) showing the importance of abdominal wall in testicular migration process. A) The abdominal wall and the gubernaculum were preserved and the testicular migration occurs in 100% of the cases in the experimental study. The arrowhead represents the intra-abdominal pression; B) The gubernaculum was sectioned and the testicular migration was completed in 70% of the cases in the experimental study; C) in the figure we can observe that authors created a defect in the anterior and the testicular migration was completed in 96% of the cases; and D) The authors performed a section in gubernaculum testis and created a defect in the abdominal wall and the testicular migration was completed in only 42% of the cases.

An interesting study has shown that intra-abdominal pressure is a supporting factor for testicular migration (4). The author performed an experiment in which defects were created in the anterior abdominal wall of animals associated or not with the section of the proximal portion of the gubernaculum (4) (Figure-3).

It became evident that there was a significant decrease in testicular migration only in cases where the abdominal wall defect was accompanied by sectioning of the gubernaculum. In cases of isolated defects in the abdominal wall the testis migrated in 96% of the cases. This experiment demonstrates that abdominal pressure would act only as an auxiliary force in testicular migration, while the gubernaculum and patency of the vaginal process would be of great importance for the orientation of the testicular path during migration (4).

## UNDESCENDED TESTIS AND PRUNE BELLY SYNDROME

**Figure 4:**
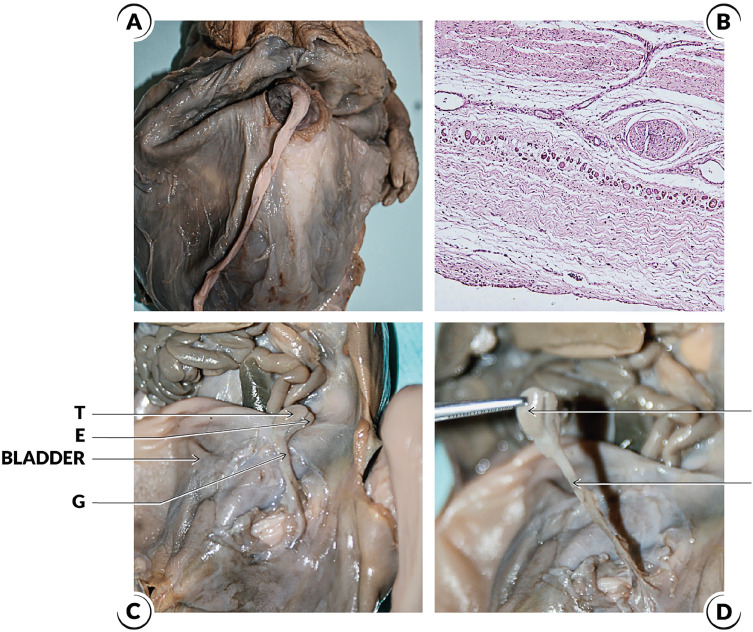
Prune Belly Syndrome cases. A) The figure shows a male fetuses with 28 weeks post conception (WPC) and Prune Belly Syndrome; the anterior abdominal wall was dissected and we can observe the typical aspect of the abdomen in this syndrome; B) Photomicrography of the same fetuses showing the histological aspect of the abdominal wall muscles with hypoplasia in Prune Belly Syndrome; HEX200; C) In this figure the abdominal wall of the Prune Belly Syndrome fetus with 28WPC was dissected and we can observe an enlarged bladder and the left testis (T) situated in abdominal position very close to the bladder, we also observe the distal insertion of the gubernaculum testis (G) and the epididymis (E); and D) In this figure the left testis was dissected in this fetus with 28WPC and Prune Belly Syndrome to show the relationship between the testis (T) and the gubernaculum (G).

Prune Belly syndrome (PBS) is a rare disorder with an incidence of 1:40,000 live births (affects men in > 95% of cases) (26). PBS is characterized by deficiency or hypoplasia of the abdominal muscles and/or malformation of the urinary tract, such as large and hypotonic bladders, dilated and tortuous ureters and bilateral cryptorchidism (24, 27) (Figure-4). The main pathogenic theory of PBS is urethral obstruction that would cause distension of the urinary tract, preventing the normal development of the abdominal musculature and the descent of the testes (24).

Urethral obstruction occurs in one-third of patients with PBS and could be the primary cause of the malformations in this syndrome (28, 29). Bilateral cryptorchidism is characteristic of Prune Belly syndrome (24, 27). The most important theories to explain bilateral cryptorchidism in this syndrome are: a) impaired contraction of the muscles of the abdominal wall; b) mechanical obstruction due to bladder distention; c) structural alteration of the inguinal canal, which hampers the passage of the testis; and d) structural alterations in gubernaculum testis (24, 27). Recently, an important paper studied the structure of gubernaculum testis in human fetuses with PBS and found alterations in the concentrations of collagen and elastic fibers and observed a small quantity of nerves both in the gubernaculums of the control group and those of the PBS group (30).

The cause of the cryptorchidism in this syndrome is unknown, but it is speculated that anatomical changes in the anterior abdominal wall hinder the increase of intra-abdominal pressure, one of the factors necessary for testicular descent. It has been speculated that the large bladder in this syndrome makes the inguinal canal extra-peritoneal, so that the gubernaculum and its contained processus vaginalis are not able to develop normally within the inguinal canal normally (24, 28, 29).

Another theory put forward to explain bilateral cryptorchidism in PBS is the structural alteration of the inguinal canal, which hampers the passage of the testis (24). Previous paper shows structural alterations in development of processus vaginalis inside the gubernaculum in Prune Belly Syndrome. These structural alterations could be one of the factors involved in cryptorchidism in Prune Belly syndrome (30). This important paper speculates that the occurrence of a mechanical obstruction or the altered intra-abdominal pressure in PBS hinders the remodeling of the gubernaculum (30).

Recent papers show that there are no important differences in development of the testes in fetuses with Prune Belly Syndrome (31). This finding suggests that bilateral cryptorchidism in PBS does not alter the testicular development and growth during the fetal period.

## UNDESCENDED TESTIS IN PATIENTS WITH ABDOMINAL WALL DEFECTS

**Figure 5:**
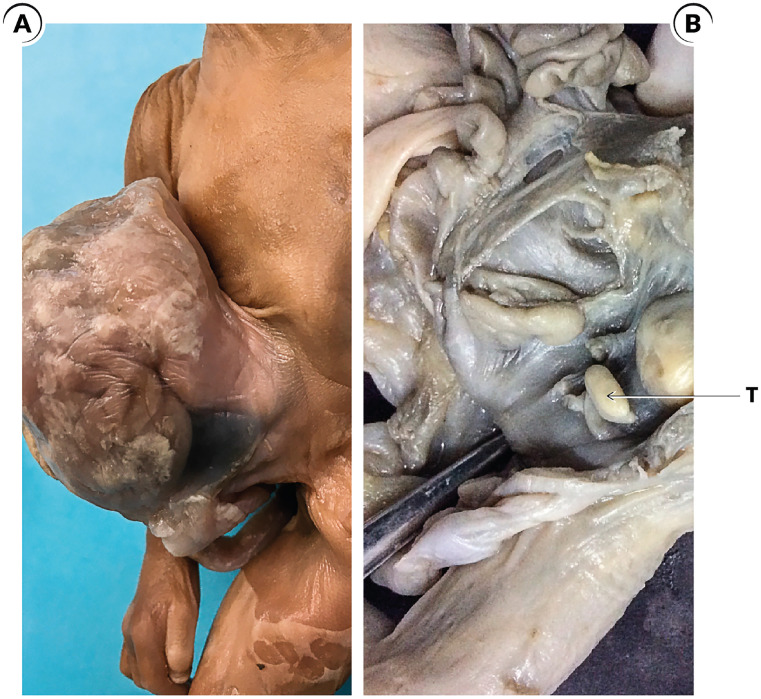
Abdominal wall defects (AWDs) cases. A) The figure shows a male fetus with 24WPC and omphalocele; and B) In a fetus with 22WPC and omphalocele we can observe the testis (T) situated in the abdominal position.

Abdominal wall defects (AWDs) are common human birth anomalies with incidence of about 1 in 2,000 newborns (32). AWDs that occur most commonly are gastroschisis and omphalocele (33) (Figure-5). Gastroschisis is a paraumbilical AWDs associated with protrusion of the abdominal content through a defect, while in omphalocele the defect is the location of the umbilicus and abdominal viscera outside the belly in a herniated sac (34, 35). Omphalocele is characterized by the failure of the physiological hernia to return to the abdominal cavity (36). On the other hand, the cause of gastroschisis is not completely elucidated, but there is evidence of an abnormality in the formation and development of the ventral body wall during embryogenesis, resulting in bowel herniation (37).

Patients with omphalocele have a high prevalence of associated anomalies, but gastroschisis is associated with malformations outside the gastrointestinal tract in around 10% of the cases, and with abnormalities related to the gastrointestinal tract in up to 25% of cases (38, 39). In fetuses with defects in the abdominal wall, the organs tend to protrude out through the abdominal opening. In most cases, two or more organs (e.g., liver, intestines and stomach) are herniated (Figure-5) (40, 41).

Koivusalo et al. (42) shows high rates of undescended testis but without correlation between the abdominal wall defect extension and the incidence of undescended testis. Previous studies show that AWDs are associated with undescended testes in around 30 to 40% of the cases. In these patients the spontaneous testicular descent occurs in about in 50% of the cases (23, 43, 44). The association of AWDs and undescended testis probably occurs by mechanical factors rather than prematurity and if the testis easily reaches the scrotum, orchidopexy can be done at the time of gastroschisis repair (23) but the primary orchiopexy should be attempted in cases of abdominal testes because the high testicular salvage rates (45–47). In cases in which the spermatic cord is not long enough to place the testis into the scrotum, mobilization and fixation at the lowest site possible resulted in better outcomes than leaving the testis in the abdomen (45–47).

## CONCLUSIONS

The abdominal pressure would be an auxiliary force in testicular migration. Patients with abdominal wall defects are associated with undescendend testis in more than 30% of the cases probably by mechanical factors and the Prune Belly Syndrome has anatomical changes in the anterior abdominal wall that hinder the increase of intra-abdominal pressure which could be the cause of cryptorchidism in this syndrome.
